# Identification of a bacteria-produced benzisoxazole with antibiotic activity against multi-drug resistant *Acinetobacter baumannii*

**DOI:** 10.1038/s41429-021-00412-7

**Published:** 2021-02-12

**Authors:** Robert W. Deering, Kristen E. Whalen, Ivan Alvarez, Kathryn Daffinee, Maya Beganovic, Kerry L. LaPlante, Shreya Kishore, Sijing Zhao, Brent Cezairliyan, Shen Yu, Margaret Rosario, Tracy J. Mincer, David C. Rowley

**Affiliations:** 1 Department of Biomedical and Pharmaceutical Sciences, College of Pharmacy, University of Rhode Island, Kingston, RI USA; 2 Department of Biology, Haverford College, Haverford, PA USA; 3 Department of Pharmacy Practice, College of Pharmacy, University of Rhode Island, Kingston, RI USA; 4 Infectious Diseases Research Program, Providence Veterans Affairs Medical Center, Providence, RI USA; 5 Octagon Therapeutics, Inc., Cambridge, MA USA; 6 Wilkes Honors College and Harbor Branch Oceanographic Institute, Florida Atlantic University, Boca Raton, FL USA

**Keywords:** Clinical pharmacology, Antibiotics

## Abstract

The emergence of multi-drug resistant pathogenic bacteria represents a serious and growing threat to national healthcare systems. Most pressing is an immediate need for the development of novel antibacterial agents to treat Gram-negative multi-drug resistant infections, including the opportunistic, hospital-derived pathogen, *Acinetobacter baumannii*. Herein we report a naturally occurring 1,2-benzisoxazole with minimum inhibitory concentrations as low as 6.25 μg ml^−1^ against clinical strains of multi-drug resistant *A. baumannii* and investigate its possible mechanisms of action. This molecule represents a new chemotype for antibacterial agents against *A. baumannii* and is easily accessed in two steps via de novo synthesis. In vitro testing of structural analogs suggest that the natural compound may already be optimized for activity against this pathogen. Our results demonstrate that supplementation of 4-hydroxybenzoate in minimal media was able to reverse 1,2-benzisoxazole’s antibacterial effects in *A. baumannii*. A search of metabolic pathways involving 4-hydroxybenzoate coupled with molecular modeling studies implicates two enzymes, chorismate pyruvate-lyase and 4-hydroxybenzoate octaprenyltransferase, as promising leads for the target of 3,6-dihydroxy-1,2-benzisoxazole.

## Introduction

*Acinetobacter baumannii* is a non-fermenting, Gram-negative member of the Gammaproteobacteria commonly implicated in nosocomial sepsis, wound infections, and ventilator-associated pneumonia [1]. The World Health Organization recognizes antibiotic resistance as one of the three greatest threats to human health, and recently classified carbapenem-resistant *A. baumannii* as one of the most urgent threats, calling for a renewed investment in antibiotic development [2]. *A. baumannii* tenacity in hospitals is enhanced both by its ability to develop antibiotic resistance and capacity to survive on surfaces, including skin, for several days [3]. *A. baumannii* can also spread via aerosolization, promoting this pathogen’s ability to easily colonize new environments [4–6]. *A. baumannii* is a member of the so-called ‘ESKAPE’ pathogens [7] and has garnered significant attention due to its propensity to cause wound infections in U.S. service members who served in Iraq and Afghanistan [8]. Alarmingly, intensive care unit patients contracting *A. baumannii* infections have a high mortality rate (>50%), complicated by the fact that new pan-drug resistant (PR) strains of *A. baumannii* with resistance to all chemotherapeutic treatment options have now emerged [1, 8–10]. Carbapenems, typically in combination with other antimicrobial agents (e.g., the polypeptide colistin) are a commonly utilized treatment option for multi-drug resistant (MDR) *A. baumannii*, but it is estimated that more than half of MDR strains are now carbapenem-resistant and colistin has significant toxicological and dosing concerns [1, 11, 12]. With the lack of new antibiotics in the drug discovery pipeline to treat Gram-negative infections, coupled with accelerated evolution of antibiotic resistance, it is imperative that new antibacterial drugs for treating *A. baumannii* infections be developed [13].

Although uncommonly reported from natural sources, benzisoxazole scaffolds are well represented in pharmacology, having shown significant importance in anti-HIV [14], antimicrobial [15–18], antipsychotic [19, 20], anti-inflammatory [17, 21, 22], antioxidation [17, 23], anticancer [23–26], anticonvulsant [27] and antidiabetic [28, 29] research. Moreover, 1,2-benzisoxazole derivatives, which include zonisamide, risperidone, paliperidone, and iloperidone, are all FDA approved and currently in use for epilepsy, mood disorders, and schizophrenia, respectively [30–33].

Herein we report the identification of 3,6-dihydroxy-1,2-benzisoxazole (**1**), a potent antibiotic against *A. baumannii* produced by a marine bacterium identified as a *Bradyrhizobium denitrificans*. A series of synthetically prepared analogs define key structural features for the antibacterial effects. We further provide evidence suggesting the mechanism of action (MOA) of 3,6-dihydroxy-1,2-benzisoxazole. In discovering 4-hydroxybenzoate (4-HB)’s ability to reverse the antibacterial property of **1**, we investigate two possible 4-HB-utilizing target enzymes, chorismate pyruvate-lyase (CPL) and 4-HB octaprenyltransferase.

## Results

### Bioassay-guided fractionation and dereplication of 3,6-dihydroxy-1,2-benzisoxazole

Crude organic extract from the exudate of *B. denitrificans* (Isolate B158) was generated and initially screened in the *p*-iodonitrotetrazolium chloride (INT) assay to assess MDR reversal potential as described [34]. This initial screening demonstrated the extract from isolate B158 potentiated (i.e., reducing the antibiotic MIC by at least 4-fold) the activity of erythromycin when tested against *E. coli* MDR strains MG1655 ΔBC/pABM, and MG1655 ΔBC/pXYM. Phylogenetic analysis of 16S rRNA gene sequence indicated that isolate B158 (Genbank Accession no. MF113387.1) was most closely related to *B. denitrificans*. Bioassay-guided fractionation of crude extract generated from 72 l of B158 microbial culture resulted in the isolation of compound **1**.

Compound **1** was isolated as an amorphous white powder. An [M − H]^−^ ion of 150.0204 using HRESIMS indicated a molecular formula of C_7_H_5_NO_3_. ^1^H NMR resonances at *δ*_H_ 7.49, 6.75, and 6.67 as well as the molecular formula suggested **1** to be 3,6-dihydroxy-benzisoxazole [35]. Because only 1 mg of compound **1** was isolated from 14.1 g of crude extract, 3,6-dihydroxy-benzisoxazole was synthesized (Fig. S1) and determined to be identical to the natural product by NMR spectroscopy (Table S1) [35]. The synthetic derivative of compound **1** showed potent antimicrobial activity against *Escherichia coli* MDR strains AG102, MG1655 ΔBC/pABM, and MG1655 ΔBC/pXYM with an MIC between 0.31 and 0.63 µg ml^−1^.

### 3,6-Dihydroxy-1,2-benzisoxazole (1) inhibits the growth of clinically relevant strains of *A. baumanii*

Compound **1** was previously reported to possess growth inhibitory effects against Gram-negative pathogenic bacteria, but not Gram-positives [36]. Because this prior testing was completed more than 30 years ago and did not include *A*. *baumannii*, we tested **1** for antibacterial activity against a panel of four clinically relevant *A*. *baumannii* strains (Table S2). Two strains (L1051 and NR-13382) were classified as carbapenem-resistant *A. baumannii* (CRAB) and as multi-drug resistant (MDR) organisms by having non-susceptibility to one or more agents in three or more antimicrobial categories using previously published guidelines [37, 38]. Minimum inhibitory concentrations (MICs) were determined to be in the range of 6.25 μg ml^−1^ to 50 μg ml^−1^ with the most potent antibacterial effects against the MDR strains NR-13382 and L1051 (6.25 and 12.5 μg ml^−1^, respectively). While the MICs for these clinical strains were measured in Muller-Hinton broth (MHB), additional MIC testing was pursued using both MHB and DM01, a defined minimal medium supplemented with sodium pyruvate as a carbon source. We hypothesized that nutrient replete media could contain compounds that antagonized the antibiotic activity of **1**, which could provide insight into compound **1**’s MOA. Interestingly, compound **1** was much more potent against multiple strains of *Pseudomonas aeruginosa, E. coli, A. baumannii*, and *Klebsiella pneumoniae* in assays using minimal media, DM01 (Table 1).

**Table 1:** MIC values for 3,6-dihydroxy-1,2-benzisoxazole (1) against Gram-negative bacteria in this study

Strain	MIC (µg ml^−1^)	
	DM01	MHB
*E. coli* ATCC 25922^TM^	0.25-0.5	>500
*E. coli* UNT 156	0.25	64
*P. aeruginosa* UCBPP14	8–16	>500
*P. aeruginosa* PA27	16	500
*K. pneumoniae* ATCC 43816^TM^	>64	>500
*K. pneumoniae* ATCC BAA-2146^TM^	1-2	>500
*A. baumannii* UNT 190	2	128
*A. baumannii* UNT 197	2	16
*A. baumannii* L1051	nt	12.5
*A. baumannii* Isolate 9, NR-13382	nt	6.25
*A. baumannii* Naval-81, NR-17786	nt	25
*A. baumannii* ATCC 19606^TM^	nt	50

*nt* not tested

### Testing of synthetic analogs of 3,6-dihydroxy-1,2-benzisoxazole

**Fig. 1:**
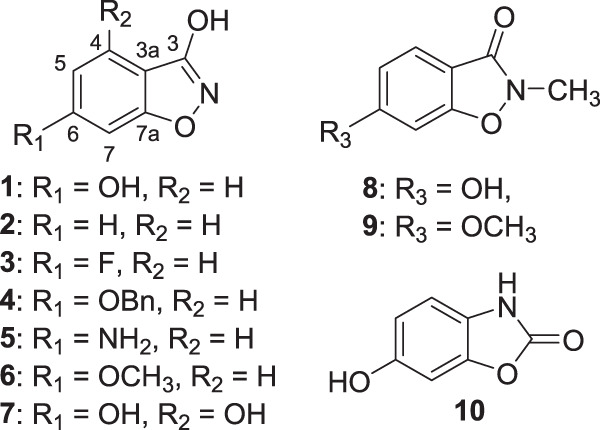
Panel of compounds tested for MICs against *A. baumannii* in SAR study

A panel of analogs was synthesized and purchased in order to probe the structural requirements necessary for the antibacterial effects of **1** as well as to potentially improve upon its potency (Fig. 1). None of the analogs (**2–10**) demonstrated increased potency against the panel of clinical *A. baumannii* strains (Table S2). However, structural requirements for optimal potency were revealed. Replacement of the hydroxyl substituent at C6 (as in **2–6**) displayed pronounced decreases in potency. Based on these data, a (H-bond) donor substituent at C6 appears to be required for antibacterial effects, with hydroxyl (**1**) as the preferred substituent over amino (**5**). Though the C6 methoxyl group (**6**) did not completely abolish activity, the dramatic decrease in potency concludes that alkyl modifications to the C6 hydroxyl group are unlikely to improve potency in this pharmacophore. Because of the preference of the hydroxyl substituent at the C6 position, an additional hydroxyl group was installed at C4 (**7**), but this abolished the antibacterial activity.

In addition to substituents on the benzene ring, the necessity of the isoxazole ring to the pharmacophore was investigated. Methylation of the nitrogen atom (**8** and **9**) led to inactive products; it is noted that this modification leads to loss of conjugation in the hydroxyl-isoxazole ring. Similarly, antibacterial activity was also completely absent from the oxazolone analog (**10**).

### 4-Hydroxybenzoic acid antagonizes the antibacterial activity of 3,6-dihydroxy-1,2-benzisoxazole

We next sought to more stringently test and better understand the mode of action of the active compound. The lower MICs observed in the minimal medium DM01 versus the replete growth medium MHB suggested that compounds in MHB might interfere with the antibiotic activity of **1**. Growth characteristics of *A. baumannii* Ab197 and *P. aeruginosa* UCBPP14 (PA14) were measured using 96-well phenotype microarray plates (PM1-PM5) purchased from Biolog, Inc. The bacteria strains were cultured in DM01 medium both with and without 2 µg ml^−1^ of compound **1** and growth was measured at 17 and 23 h. These experiments revealed that 4-hydroxybenzoic acid (4-HB) and 4-hydroxybenzaldehyde antagonized the antibacterial effects of **1**. To further examine this effect, PA14 was cultured in DM01, both with and without 16 µg ml^−1^ of **1** and 2-fold serial dilutions of 4-HB and 4-hydroxybenzaldehyde (Fig. S2). Both 4-hydroxybenzoic acid and 4-hydroxybenzaldehyde antagonized the growth inhibitory effects of **1** at the three lowest concentrations tested (62.5–250 µg ml^−1^).

### Molecular docking of 3,6-dihydroxy-1,2-benzisoxazole onto *A. baumannii* CPL homology model

**Fig. 2:**
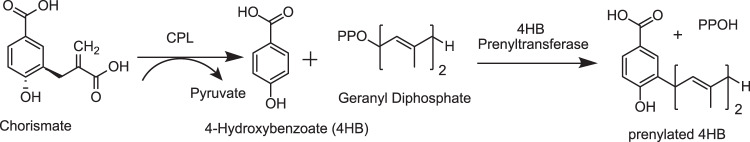
Formation and utilization of 4-hydroxybenzoate by bacteria. Conversion of chorismate to 4-hydroxybenzoate (4HB) by chorismate pyruvate-lyase (CPL) in *E. coli*, followed by enzymatic synthesis of prenylated-4-hydrobenzoate catalyzed by 4HB octaprenyltransferase

**Fig. 3:**
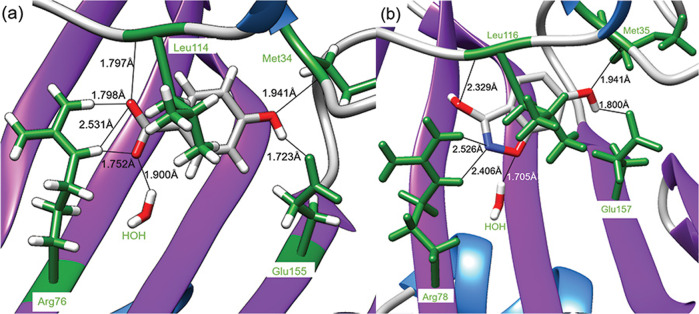
Binding conformation of 4HB in *E. coli* CPL (PDB 1FW9) and compound **1** in *A. baumannii* (GenBank: SST05187.1) model. Hydrogen bonds (black lines) between ligand (gray) and protein residues (green) are labeled. **a** Binding of 4HB to *E. coli* CPL crystal structure (PDB:1FW9). **b** Molecular docking of compound **1** onto *A. baumannii* homology model under solvated (water) environment

The phenotypic array highlighted the antagonistic effect of 4-HB against **1**, suggesting our benzisoxazole antibiotic may be targeting bacterial metabolic processes that involve 4-HB (Fig. 2). To examine if compound **1** has the potential to inhibit chorismate pyruvate-lyase (CPL), involved in the formation of 4-HB, we performed an in silico molecular docking study. In the absence of a crystal structure for *A. baumannii* CPL, *E. coli* (UniProt P26602) CPL was used to create a homology model. Superposition of the *A. baumannii* model with *E. coli* template shows a root-mean-square distance (RMSD) value of 0.780 Å (Fig. S3), indicating a good fit to the model. Molecular docking under the solvated environment finds that compound **1** exhibits a favorable predicted binding affinity of −5.8 kcal mol^−1^ (Table S3) and interacts with the primary site of *A. baumannii* model (Figs. 3 and S4). The binding of 4-hydroxybenzoate to *E. coli* CPL and compound **1** in *A. baumannii* involves five hydrogen bonds with four conserved residues (Arg76/Arg78, Glu155/Glu157, and peptide amide of Met34/Met35 and Leu114/Leu116) (Fig. 3).

## Discussion

1,2-benzisoxazoles are bicyclic, heteroaromatic structures found in many approved drugs, are able to cross the blood-brain barrier, and have been classified as privileged structures for drug design for central nervous system disorders and other therapeutic areas [39, 40]. The heterocyclic ring structure of **1** shares similarity with the clinically used antibiotics cycloserine and linezolid. Interestingly, cycloserine was inactive against our panel of *A. baumannii* strains (data not shown). Oxazolidinone antibiotics are only effective against Gram-positive pathogens, and match the ring structure of the inactive **10**, further supporting the highly specific nature of the 1,2-benzisoxazole pharmacophore for Gram-negative pathogens. Compound **1** was previously reported to possess potent growth inhibition of Gram-negative bacteria including *E. coli*, *Proteus* spp., *Salmonella* spp., *K. pneumoniae*, *Enterobacter* spp., and *Serratia marcescens*, as well as low toxicity with an LD_50_ of over 1500 mg in mice [35, 36]. Previous work indicated no inhibition was observed with the Gram-positive bacteria *Staphylococcus aureus, Bacillus* spp., *Micrococcus* sp., *Corynebacterium* sp., or the Gram-negative *Pseudomonas* spp. [35, 36].

From the panel of analogs tested, it appears that the structural features in the natural product **1** are necessary components of the antibiotic pharmacophore. A hydrogen bond donor is necessary at C6, and a hydroxyl is preferred over an amino group. Modifications to the heteroaromatic ring were found to be detrimental. Further optimization of the structure for antibacterial effects against *A. baumannii* would include determining if the 3-position is modifiable, as well as exploring other positions on the benzene ring for substitution.

Our results demonstrate that the antibacterial effect of **1** on *A. baumannii* can be reversed by supplementing the growth medium with 4-hydroxybenzoate (4-HB) and, to a lesser extent, by 4-hydroxybenzaldehyde. 4-HB is a precursor of ubiquinone biosynthesis for the electron transport chain and aerobic growth [41–43]. Connecting the antagonist effect of 4-HB with possible mechanisms of action, we have formulated two hypotheses pertaining to two target enzymes, CPL and 4-HB octaprenyltransferase, both of which are involved in the production and transformation of 4-HB (Fig. 2).

The production of 4-HB differs across animals and bacteria. Mammals synthesize 4-HB from tyrosine [41, 43], while Gram-negative bacteria produce 4-HB from chorismate, a product of the shikimate pathway [41, 44, 45]. In the first step of ubiquinone biosynthesis, bacterial CPL, the sole producer of 4-HB [46], irreversibly converts chorismate into 4-HB and pyruvate [46–48] (Fig. 2). The catalytic cycle of bacterial CPL involves a solvent accessible secondary binding site interconnected to an internal primary active site [49, 50], where product 4-HB exhibits a ten-fold higher binding affinity than chorismate [47, 49–51]. This product inhibition mechanism allows 4-HB to limit ubiquinone production via a negative feedback pathway [49, 51, 52] and stabilize CPL in the absence of chorismate-binding [50]. When chorismate binds at the secondary site, this entry encourages the release of 4-HB from the primary site, where chorismate subsequently enters and is converted into 4-HB and pyruvate [50]. Pyruvate leaves, while 4-HB remains bound to CPL for structural stability [50, 51]. The CPL enzymes of four Gram-negative bacteria (*E. coli*, *A. baumannii*, *K. pneumoniae*, *S. marcescens*), which are all susceptible to **1** [35, 36], share between 83–100% amino acid identity among the active site residues (Fig. S5), further bolstering a possible benzisoxazole-mediated binding to CPL. Furthermore, the lack of CPL in mammals may account for **1**’s low toxicity to mice [35, 36].

Our molecular docking results provide additional support of the CPL inhibition hypothesis, where compound **1** interacts with the primary site of CPL (Figs. 3 and S4). The five hydrogen-bonds and four conserved residues observed in the binding of 4-HB in *E. coli* are likewise maintained in compound **1**’s interaction with *A. baumannii* model, thereby indicating a similar binding conformation. Overall, the conserved binding interactions suggest that **1** may be a competitive product inhibitor of CPL, as a sufficient concentration of **1** may outcompete 4-HB at the primary binding site. The absence of 4-HB-binding may disrupt CPL’s catalytic cycle [50, 51] and prevent the production of 4-HB, thus arresting ubiquinone pathway [46–48] and aerobic bacterial growth [41, 42].

Following CPL’s production of 4-HB, membrane-bound 4-HB octaprenyltransferase (UbiA) prenylates 4-HB [41, 47, 48, 53], opening up yet a second speculation for compound **1**’s mechanism of action. 4-HB octaprenyltransferase is a membrane-bound enzyme in the ubiquinone biosynthesis pathway, encoded by the *ubiA* gene in bacteria and by the *COQ2* gene in mammals [41]. In the first step of the ubiquinone biosynthesis pathway, the bisubstrate enzyme binds both 4-HB and geranyl diphosphate to prenylate the meta position of 4-HB to yield 3-polyprenyl-4-hydroxybenzoate (Fig. 2) [54]. A second hypothesis is then that **1** competitively inhibits 4-HB binding to UbiA, thereby preventing ubiquinone biosynthesis. Support for this second possibility comes from three main conditions: (i) 4-HB octaprenyltransferase is a promiscuous, non-specific enzyme towards both its substrates [41, 55] (ii) the necessity of a hydrogen bond donor at C6 for transfer of hydrogen to the negatively charged Asp191, which is theorized to result in the enhancement of the negative partial charge at the meta-position and hence activation of the benzene ring [54] (iii) the necessity of a functional group that is able to hydrogen bond at the position para to C6 in order to interact with Arg72 [54]. Both Asp191 and Arg72 are active site residues. Compound **1** satisfies both condition ii and iii and may mimic 4-HB, hindering prenylation either by blocking the active site or by getting prenylated itself. In the former case, ubiquinone cannot be formed without the prenyl tail. In the latter case, the prenylated products might not be able to proceed through the ubiquinone biosynthesis pathway due to the specificity of subsequent enzymes [56, 57]. A sequence alignment with *E. coli’s* UbiA amino acid sequence (Fig. S6) demonstrated that Asp191 is conserved across three bacteria and two mammalian species, while Arg72 is conserved across *E. coli, A. baumannii, P. aeruginosa* and *Mus musculus* but not in humans. Hence, the active site Arg72 could enhance the specificity of **1**’s interaction in bacteria and lower mammals.

The ability to bind to CPL would also account for the reduced activity of the analogs **2**–**9**. Compounds **2–4, 6**, and **9** all lack a C6 hydroxyl group and therefore would not enable H-bonding from C6 to the active site Asp191. Compounds **8** and **9** would lack the ability to form an H-bond from the C3 position to Arg72. With regard to compound **7**, it has been previously shown that substitutions to 4-HB at both ortho positions are not accepted as substrates for 4-HB octaprenyltransferase [55]. Compound **5**, which substitutes an amine in place of the C6 hydroxyl group, retained modest activity, perhaps because it may still H-bond with the active site residue Asp191. 4-HB octaprenyltransferase has previously been shown to tolerate a switch from hydroxy to an amino group in the 4-HB substrate [55].

It is interesting that the antibiotic effects of compound **1** are intensified under minimal media conditions. Marine bacteria frequently compete under minimal nutrient conditions for a good part of their life cycle [58]. Future studies to decipher the selective pressures that compound **1** are expressed under could yield more information as to the natural history of this molecule and allelopathic pathways in nature.

Narrow spectrum antibacterial drugs are becoming more desirable due to advances in diagnostic tests and changes to the regulatory environment [59]. Resistance is expected to develop more slowly to narrow-spectrum antibiotics and collateral damage to the gut microbiome, such as antibiotic-associated colitis, is less likely [60]. Compound **1** was more active against *A. baumannii* strains than other Gram-negative bacteria when measured using standard MIC assays using Mueller-Hinton broth. Coupled with the current paucity of drugs available to treat MDR *A. baumannii* infections, we suggest that further studies to advance the development of 1,2-benzisoxazole antibiotics are warranted.

## Materials and methods

### General experimental procedures

NMR experiments were conducted using an Agilent NMRS 500 MHz spectrometer with (CD_3_)_2_SO (referenced to residual DMSO at *δ*_H_ 2.50 and *δ*_C_ 39.5) or CD_3_OD (referenced to residual CH_3_OH at *δ*_H_ 3.31 and *δ*_C_ 49.0) at 25 °C. High Resolution Electrospray ionization mass spectra (HRESIMS) were acquired using an AB Sciex TripleTOF 4600 spectrometer in the negative ion mode. Flash chromatography was completed with a Combiflash Rf200 equipped with a 40 g silica gel RediSepRf High Performance Gold column (Teledyne ISCO). HPLC experiments were performed on a Hitachi Elite LaChrom system equipped with a diode array detector (DAD) model L-2450, pump L-2130, and autosampler L-2200. Semi-preparative HPLC purifications were accomplished with a Waters XBridge Prep C18 5 µm, 10 × 250 mm column at 4.5 ml min^−1^.

### Microbial isolation and identification

A single comb jelly, *Mnemiopsis leidyi*, was obtained via collection by dip net at the surface from Eel Pond, Woods Hole, MA (41° 31’ 32” N, 70° 40’ 12” W). The digestive contents were stamped onto a tryptone seawater agar (TSW; 1 g l^−1^ tryptone; 1 l of 75:25 natural seawater:Milli-Q water; 15 g agar) plate and cultivated at 23 °C for three days. A single semi-transparent colony was transferred onto a fresh TSW plate to obtain a pure isolate of strain B158. The isolated strain B158 was identified to be *B. denitrificans* on the basis of 97.3% similarity in the 16S rRNA gene sequence (2370 nucleotides; Genbank Acc. No. MF113387.1) to *B. denitrificans* NBRC 105663 (Genbank Acc. No. AB682257.1). Pure stocks were frozen back in 10% DMSO and stored at −80 °C until use.

### Bacteria culture and extract production

A starter culture of B158 was prepared by inoculating 6 ml of TSW media and with 100 µl of frozen culture and incubated at 23 °C, 100 rpm for 3 days. Large batch cultures to scale up growth consisted of 1.5 l of TSY (1 g l^−1^ tryptone, 1 g l^−1^ yeast extract, 1 l of 75:25 natural seawater: Milli-Q water) media inoculated with 1.5 ml of ‘starter’ culture and grown at 100 rpm for 8 days at 23 °C. Twenty-four hours before culture filtration (day 7), 20 ml of 1:1 mixture of sterile, washed Amberlite XAD-7 and XAD-16 resin was added to the cultures. On the eighth day, the resin was filtered from the bacterial culture under vacuum, desalted by rinsing with Milli-Q water, and allowed to dry overnight at room temperature.

Metabolites were eluted from the resin first in 100 ml of (1:1) methanol/dichloromethane, followed by 100 ml of methanol. This extract was then dried under vacuum centrifugation (ThermoSavant). Dried extracts were stored at −80 °C until bacterial susceptibility assays. In sum, 72 l of B158 culture were processed as described above and yielded 14.5 g of crude extract. The crude extract was subjected to silica gel column chromatography with a step gradient of 100% isooctane, 4:1 isooctane/ethyl acetate; 3:2 isooctane/ethyl acetate, 2:3 isooctane/ethyl acetate, 1:4 isooctane/ethyl acetate, 100% ethyl acetate, 1:1 ethyl acetate/methanol, and 100% methanol, yielding eight fractions. Active constituents, as determined with the INT assay, eluted in 100% ethyl acetate and 1:1 ethyl acetate/methanol (fractions 6 and 7) were combined (totaling 2374 mg) and further fractionated by SPE/ENVI-18 sep pak with a step gradient of 19:1 water/acetonitrile, 4:1 water/acetonitrile, 13:7 water/acetonitrile, 1:19 water/acetonitrile, and 100% acetone. All solvents with the exception of acetone were acidified with 0.1% formic acid. Active constituents eluted in 4:1 water/acetonitrile and 13:7 water/acetonitrile. These fractions were combined (totaling 698.4 mg) and further chromatographed using a gradient of acetonitrile (0.1% formic acid) and water (0.1% formic acid) by semipreparative HPLC carried out on an Agilent 1200 series equipped with an autosampler, diode array detector, quaternary pump, and 96-well plate fraction collector with a Phenomenex Luna 5 µm C_18_(2), 100 Å, LC column (250 × 10 mm) as the stationary phase with a flow rate of 4 ml min^−1^. Chromatography methods were as follows: begin at 5% acetonitrile and ramp to 40% acetonitrile over 17 min, ramp to 95% acetonitrile over 10 s and hold for 7 min, return to 5% acetonitrile over 10 s and hold for 4 min at starting conditions. Active constituents eluted between 30–32% acetonitrile with the resulting activity spread over two wells of a 96-well plate totaling 32.4 mg. The final purification of active fractions was achieved by using a gradient of methanol (0.1% formic acid) and water (0.1% formic acid) by analytical HPLC (Phenomenex Kinetex 2.6 µm C18 100 Å, LC column (150 × 2.1 mm) as the stationary phase with a flow rate of 0.2 ml min^−1^ and UV detection at 276 nm. Chromatography methods were as follows: being at 5% methanol and hold for 5 min, ramp to 40% methanol over 11 min, hold at 40% methanol for 1 min, ramp up to 95% methanol over 10 s, hold at 95% methanol for 3 min, then immediately revert to starting conditions and hold for 3 min. Compound 1 (1 mg) eluted at 13.3 min to yield 6-hydroxy-1,2 benzoxazol-3(2*H*)-one.

### 3,6-dihydroxy-1,2-benzisoxazole (1)

The synthesis shown in Supplementary Fig. 1 is based on one previously published [61]. Hydroxylamine hydrochloride (2.50 g, 36.0 mmol) was added to a stirring solution of KOH (2.69 g, 47.9 mmol) in water (30 ml) at room temperature. Methyl 2,4-dihydroxy-benzoate (1.01 g, 6.0 mmol) dissolved in 1,4 dioxane (5 ml) was added dropwise to the hydroxylamine solution and stirred for 24 h at room temperature. After removing the 1,4 dioxane in vacuo, the pH was adjusted to 1 using 3 N HCl and extracted with ethyl acetate (3 × 50 ml). The organic layers were combined and dried over Na_2_SO_4_, filtered and concentrated in vacuo. The resultant residue was purified by silica gel flash chromatography using a gradient of 20–100% ethyl acetate/hexanes to afford 2,4-dihydroxy-benzohydroxamic acid (765.7 mg, 76% yield).

2,4-dihydroxy-benzohydroxamic acid (161.4 mg, 0.96 mmol) was dissolved in anhydrous tetrahydrofuran (10 ml) under N_2_. Dry 1,1′-carbonyldiimidazole (CDI) (489.2 mg, 3.0 mmol) was added to the mixture and heated at reflux conditions for 30 min. Et_3_N (210 μl, 1.5 mmol) was then added, and the reaction proceeded at reflux for 18 h. The reaction mixture was then left to cool to room temperature, and THF was removed in vacuo. The resulting residue was reconstituted in 1 N HCl and extracted with EtOAc (3 × 50 ml). The combined organic layers were dried over Na_2_SO_4_, filtered and concentrated. The resulting residue was purified by silica gel flash chromatography using a gradient of 30–50% ethyl acetate with 0.1% formic acid (FA)/hexanes to afford 3,6-dihydroxy-1,2-benzisoxazole (107.7 mg, 74% yield, overall yield 56%). Prior to bioassay, this product was further purified by HPLC with an isocratic method of 20% MeOH in H_2_O with 0.1% FA to afford 4.8 mg of **1** (white powder). ^1^H NMR (500 MHz, CD_3_OD) *δ* 6.70 (1H, d, *J* = 1.9), 6.78 (1H, dd, *J* = 1.9, 8.7), 7.50 (1H, d, *J* = 8.6). ^13^C NMR (125 MHz, CD_3_OD) *δ* 96.2, 108.2, 114.5, 123.6, 163.2, 166.9, 167.6. HRMS calcd for C_7_H_4_NO_3_: 150.0191. Found [M − H] 150.0206.

### 3-hydroxy-1,2-benzisoxazole (2)

Compound **2** was prepared from commercially available methyl salicylate (methyl 2-hydroxy-benzoate) according to the scheme used to prepare **1**. **2** was further purified by semi-preparative HPLC using an isocratic method of 50% MeOH in H_2_O with 0.1% FA to afford 6.0 mg of **2** (white powder). ^1^H NMR (500 MHz, (CD_3_)_2_SO) *δ* 7.31 (1H, t, *J* = 7.0), 7.54 (1H, d, *J* = 8.2), 7.59 (1H, t, *J* = 7.9), 7.75 (1H, d, *J* = 8.0), 12.45 (1H, br s). ^13^C NMR (125 MHz, (CD_3_)_2_SO) *δ* 110.0, 114.6, 121.4, 122.9, 130.5, 163.0, 165.4. HRMS calcd for C_7_H_4_NO_2_: 134.0242. Found [M − H] 134.0251.

### 3-hydroxy-6-fluoro-1,2-benzisoxazole (3)

Compound **3** was prepared from commercially available methyl 2-hydroxy-4-fluoro-benzoate according to the scheme used to prepare **1**. **3** was further purified by semi-preparative HPLC using the method: 12 min gradient from 55–100% MeOH in H_2_O with 0.1% FA to yield 9.1 mg (white powder). ^1^H NMR (500 MHz, CD_3_OD) *δ* 7.09 (1H, td, *J* = 1.5, 9.1), 7.22 (1H, dd, *J* = 1.5, 9.1), 7.70 (1H, dd, *J* = 5.2, 8.9). ^13^C NMR (125 MHz, CD_3_OD) *δ* 98.3 (d, *J* = 27.7), 112.8, 113.0 (d, *J* = 25.7), 123.9 (d, *J* = 11.5), 165.7 (d, *J* = 14.4), 166.3 (d, *J* = 249.0), 167.0. HRMS calcd for C_7_H_3_FNO_2_: 152.0148. Found [M − H]: 152.0271.

### 3-hydroxy-6-(benzyloxy)-1,2-benzisoxazole (4)

Commercially available methyl 2,4-dihydroxy-benzoate (1.0 g, 5.92 mmol) was dissolved in 10 ml acetone and treated with K_2_CO_3_ (1.0 g, 7.24 mmol) and NaI (0.25 g, 1.67 mmol). The resulting suspension was stirred for 10 min under N_2_ and then benzyl bromide (700 μl, 5.85 mmol) was added dropwise. The reaction mixture was stirred for 24 h at ambient temperature. After removing the acetone in vacuo, the resulting residue was resuspended in H_2_O and extracted with EtOAc (4 × 40 ml). The organic layers were combined, dried over Na_2_SO_4_, and concentrated in vacuo. This residue (1.8 g) was purified by silica gel flash chromatography using a gradient of 10–100% EtOAc in hexanes to afford methyl 2-hydroxy-4-benzyloxy-benzoate (687.4 mg, 45.5% yield) as white crystals. Methyl 2-hydroxy-4-benzyloxy-benzoate was then subject to the synthetic steps used to obtain **1**, which afforded **4**, which was further purified by semi-preparative HPLC using an isocratic method of 50% MeOH in H_2_O with 0.1% FA. This obtained 11.0 mg (white powder). ^1^H NMR (500 MHz, (CD_3_)_2_SO) *δ* 5.17 (2H, s), 6.94 (1H, d, *J* = 8.7), 7.15 (1H, s), 7.34 (1H, t, *J* = 7.4), 7.40 (2H, t, *J* = 7.4), 7.46 (2H, d, *J* = 7.4), 7.60 (1H, d, *J* = 8.6). ^13^C NMR (125 MHz, (CD_3_)_2_SO) *δ* 69.9, 94.5, 108.3, 113.4, 122.0, 127.9 (2 C), 128.1, 128.5 (2 C), 136.5, 161.1, 164.9, 165.7. HRMS calcd for C_14_H_10_NO_3_: 240.0661. Found [M − H] 240.0564.

### 3-hydroxy-6-amino-1,2-benzisoxazole (5)

Compound **5** was prepared from commercially available methyl 2-hydroxy-4-amino-benzoate according to the scheme used to prepare **1** with minor modification. The ring-closing step was accomplished with CDI/MeCN instead of CDI/THF/Et_3_N as used for **1**. The resulting mixture was purified using semi-preparative HPLC with a gradient method from 10–55% MeOH in H_2_O with 0.1% FA. This afforded 1.1 mg of **5** (white powder). ^1^H NMR (500 MHz, (CD_3_)_2_SO) *δ* 5.80 (2H, s), 6.39 (1H, d, *J* = 1.4), 6.50, (1H, dd, *J* = 1.5, 8.4), 7.30 (1H, d, *J* = 8.4). ^13^C NMR (125 MHz, (CD_3_)_2_SO) *δ* 91.2, 103.6, 111.8, 121.8, 152.3, 165.6, 165.9. HRMS calcd for C_7_H_5_N_2_O_2_: 149.0351. Found [M − H] 149.0323.

### 3-hydroxy-6-methoxy-1,2-benzisoxazole (6)

Compound **6** was purchased commercially (Sigma–Aldrich).

### 3,4,6-trihydroxy-1,2-benzisoxazole (7)

Compound **7** was prepared from commercially available methyl 2,4,6-trihydroxy-benzoate according to the scheme used to prepare **1**. **7** was further purified from a mixture by semi-preparative HPLC with a gradient method of 25–100% MeOH in H_2_O with 0.1% FA. This afforded 6.8 mg of **7** (white powder). ^1^H NMR (500 MHz, (CD_3_)_2_SO) *δ* 6.08 (1H, d, *J* = 1.3), 6.14 (1H, d, *J* = 1.3), 10.22 (3H, br s). ^13^C NMR (125 MHz, (CD_3_)_2_SO) *δ* 86.8, 96.6, 97.3, 153.8, 161.9, 165.2, 166.6. HRMS calcd for C_7_H_4_NO_4_: 166.0140. Found [M − H] 166.0151.

### *N*-methyl-6-hydroxy-1,2-benzisoxazol-3(2*H*)-one (8) and *N*-methyl-6-methoxy-1,2-benzisoxazol-3(2*H*)-one (9)

Compounds **8** and **9** were prepared from **1** in a one-step synthesis and purification. **1** (46 mg, 0.33 mmol) was dissolved in dry MeCN (4 ml), and K_2_CO_3_ (91 mg, 0.66 mmol) was suspended in the solution. Suspension was stirred rapidly under N_2,_ and excess CH_3_I (260 μl, 4.2 mmol) was added dropwise and stirred for 18 h at room temperature. The reaction was quenched by removing MeCN in vacuo, and the resulting residue was dissolved in 1 N HCl. This solution was extracted with EtOAc (3 × 30 ml), and the combined organic layers were dried over Na_2_SO_4_, filtered and concentrated in vacuo. The resulting products were purified by semi-preparative HPLC using a 12 min gradient from 35–80% MeOH in H_2_O with 0.1% FA to afford **8** (12.1 mg, white powder) and **9** (8.5 mg, white powder).

Compound **8** ^1^H NMR (500 MHz, (CD_3_)_2_SO) *δ* 3.46 (3H, s), 6.68 (1H, d, *J* = 1.6), 6.57 (1H, dd, *J* = 1.6, 8.4), 7.55 (1H, d, *J* = 8.4), 10.73 (1H, br s). ^13^C NMR (125 MHz, (CD_3_)_2_SO) *δ* 33.3, 95.3, 106.8, 113.4, 124.7, 162.3, 163.4, 164.2. HRMS calcd for C_8_H_8_NO_3_: 166.0504. Found [M + H]: 166.0216.

Compound **9** ^1^H NMR (500 MHz, (CD_3_)_2_SO) *δ* 3.51 (3H, s), 3.86 (3H, s), 6.89 (1H, dd, *J* = 2.1, 8.6), 7.05 (1H, d, *J* = 2.0), 7.64 (1H, d, *J* = 8.6). ^13^C NMR (125 MHz, (CD_3_)_2_SO) *δ* 33.2, 56.1, 93.8, 108.1, 113.2, 124.5, 162.2, 163.7, 164.5. HRMS calcd for C_9_H_10_NO_3_: 180.0661. Found [M + H]: 180.0341.

### 6-hydroxy-2-benzoxazolinone (10)

Compound **10** was purchased commercially (abcr GmbH).

#### Biological assays

##### Media

Luria Bertani (LB) medium and cation-adjusted Mueller-Hinton broth (MHB) were purchased from Sigma-Aldrich. DM01 medium contains 25 mM potassium phosphate, pH 6 (3.3 mM K_2_HPO_4_, 21.7 mM KH_2_PO_4_), 1 mM MgSO_4_, 100 µM CaCl_2_, 75 mM sodium pyruvate, 1 g l^−1^ NH_4_Cl.

##### Bacterial susceptibility determinations

Antimicrobial activity of marine bacterial extracts, semi-purified fractions, and pure compounds were evaluated by whole-cell assays with *E. coli* isolates expressing one of three archetype RND efflux pumps (AcrAB-TolC, MexAB-OprM, and MexXY-OprM) that are known to contribute to antibiotic resistance in Enterobacteriaceae and *P. aeruginosa* clinical isolates. Each of the three *E. coli* isolates overexpressing RND efflux pumps were used in 96-well plate-based assays to determine bacterial susceptibility of B158 crude extract or fraction tested in duplicate at 1 mg ml^−1^ and determined by rapid *p*-iodonitrotetrazolium chloride colorimetric assay as described in [34]. Minimum inhibitory concentrations (MICs; defined as the lowest concentration that results in no visible growth) of the synthetically derived compound **1** was determined using a 2-fold standard microdilution method in Muller-Hinton broth (MHB) in microtiter plates as defined by the National Committee for Clinical Laboratory Standards [62] for each of the three RND overexpressing *E. coli* strains.

##### Pathogen strains and MIC testing

*A. baumannii* UNT190-1, *A. baumannii* UNT197-1, and *E. coli* UNT156-1 are clinical isolates kindly provided by Dr. William Weiss at North Texas University. *P. aeruginosa* strain UCBPP-PA14 was kindly provided by Dr. Frederick M. Ausubel. *E. coli* EC2 ((Migula) Castellani and Chalmers; ATCC 25922^TM^, *K. pneumoniae* ATCC 43816^TM^ and ATCC BAA-2146^TM^, and *A. baumannii* ATCC 19606^TM^ were obtained from the American Type Culture Collection. *A. baumannii* L1051 was obtained from a patient skin sample at Rhode Island Hospital (Providence, RI) and is maintained in the LaPlante laboratory. *A. baumannii*, Isolate 9, NR-13382 and *A. baumannii*, Strain Naval-81, NR-17786 are human blood isolates obtained from the Biodefense and Emerging Infection Research Resources Repository (BEI) Resources, NIAID, NIH. L1051 and NR-13382 are considered multi-drug resistant organisms (MDROs; non-susceptibility to one agent in three antimicrobial classes), according to previously described definitions [37, 38]. Minimum inhibitory concentrations (MICs) were determined in duplicate by broth microdilution in accordance with Clinical Laboratory Standards Institute (CLSI) standards [63, 64].

##### Phenotype microarray experiments

Five different types of 96-well phenotype microarray plates (PM1-PM5) were purchased from Biolog, Inc. A 2 ml overnight bacteria culture (*A. baumannii* strain UNT197 or *P. aeruginosa* UCBPP14) in LB was grown in a shaking incubator at 37 °C. The culture was pelleted by centrifugation, washed, and resuspended in the same volume of phosphate-buffered saline (PBS). DM01 medium with or without 2 µg ml^−1^ of **1** were each inoculated with 1/1000 volume of the resuspended culture. 100 µl of the inoculated medium was added to each well of the phenotype microarray plates. Plates were covered and incubated at 37 °C. Plates were transferred to a Spectramax i3x plate reader (Molecular Devices) for measurement of OD_600_ after 17 and 23 h.

##### Targeted antibiotic antagonism experiments (*P. aeruginosa* UCBPP14)

A 2 ml overnight culture of *P. aeruginosa* UCBPP14 in LB was grown in a shaking incubator at 37 °C. The culture was pelleted by centrifugation, washed, and resuspended in the same volume of phosphate-buffered saline (PBS). DM01 medium was supplemented with 2-fold serial dilutions of 4-hydroxybenzoic acid or 4-hydroxybenzaldehyde (Sigma-Aldrich) both with and without 16 µg ml^−1^ of **1**, then inoculated with 1/1000 volume of the bacteria suspension. The cultures were incubated at 37 °C, and OD_600_ was measured at 17 h.

##### Sequence alignment and homology modeling of CPL

The amino acid sequence of *A. baumannii* chorismate pyruvate-lyase (CPL) (GenBank: SST05187.1) was aligned with template *E. coli* CPL (PDB 1FW9), which has 85% query cover, E-value of 3 ×10^−41^, and 50.35% identity. Sequence alignment was done using MUSCLE [65]. The solvent-free *A. baumannii* homology model was created by Modeller 9.24 and refined using Modeller 9.2’s salign_2d [66, 67]. Homology model with GA341 value of 1.00 and the most negative DOPE was selected [66]. The homology model was quality assessed with PROCHECK [68]. To evaluate structural similarity in root-mean-square-deviation (RMSD), the homology model was superimposed onto the template structure using UCSF Chimera’s MatchMaker [69]. To create solvated *A. baumannii* model, water molecules of the *E. coli* CPL template were combined with the superimposed *A. baumannii* model using UCSF Chimera [69, 70].

##### Molecular docking of compound 1

Compound **1** and 4-HB were docked in *A. baumannii* (GenBank: SST05187.1) homology model using UCSF Chimera and AutoDock Vina [70, 71]. A grid box was used to define the search parameter [70, 71]. Ten protein-ligand models were produced and ranked by docking scores. The most negatively scored protein-ligand model containing >3 H-bonds was selected. H-bonds and Van der Waals interactions were predicted using FindHBond and Find Clashes/Contacts function of UCSF Chimera [70, 72].

## Supplementary information

Supplemental Material
