# Supplementary material for: Identification of a bacteria-produced benzisoxazole with antibiotic activity against multi-drug resistant Acinetobacter baumannii
Source: J Antibiot (Tokyo). 2021 Feb 12;74(6):370–80. doi: 10.1038/s41429-021-00412-7 (PMC7879144; doi:10.1038/s41429-021-00412-7)
Supplement: Supplementary file 1 — Supplemental Material [file 41429_2021_412_MOESM1_ESM.docx]

**Supplemental Information**

Identification of a Bacteria-Produced Benzisoxazole with Antibiotic Activity Against Multi-Drug Resistant Acinetobacter baumannii

Robert W. Deering^a.†^, Kristen E. Whalen^b, †, #^, Ivan Alvarez^a^, Kathryn Daffinee^c,d^, Maya Beganovic^c,d^, Kerry L. LaPlante^c,d^, Shreya Kishore^b^, Sijing Zhao^b^, Brent Cezairliyan^e^, Shen Yu^e^, Margaret Rosario^a^, Tracy J. Mincer^f,#^, and David C. Rowley^a,#^

^a^Department of Biomedical and Pharmaceutical Sciences, College of Pharmacy, University of Rhode Island, Kingston, Rhode Island, USA

^b^Department of Biology, Haverford College, Haverford, Pennsylvania, USA

^c^Department of Pharmacy Practice, College of Pharmacy, University of Rhode Island, Kingston, Rhode Island, USA

^d^Infectious Diseases Research Program, Providence Veterans Affairs Medical Center, Providence, Rhode Island, USA

^e^Octagon Therapeutics, Inc., Cambridge, MA, USA

^f^Wilkes Honors College and Harbor Branch Oceanographic Institute, Florida Atlantic University, Boca Raton, Florida, USA

^#^Address correspondence to Kristen E. Whalen, kwhalen1@haverford.edu; Tracy Mincer tmincer@fau.edu; David. C. Rowley, drowley@uri.edu

^†^R.W.D. and K.E.W. contributed equally to this work.

The authors declare no financial interests

Contains additional

Supplemental Figures (1 - 6)

Supplemental Tables (1 - 3).

**Supplemental Figures**

**Supplemental Figure 1.** Schematic for synthesis of 3,6-dihydroxy-benzisoxazole (**1**). *a* – H_2_NOH:HCl/KOH in Dioxane/H_2_O; ­*b* – CDI/Et_3_N/THF reflux.

**
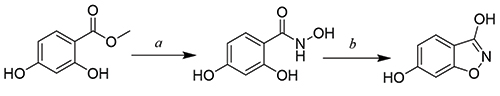
**

**Supplemental Figure 2.** Protective effect of 4-HB (a) and 4-hydroxybenzaldehyde (b) against compound **1** susceptibility in *P. aeruginosa* UCBPP14 (PA14) at 17 h post treatment. Growth curves were each performed under increasing concentration of 4-HB, 4-hydroxybenzaldehyde, and with or without compound **1**.

**
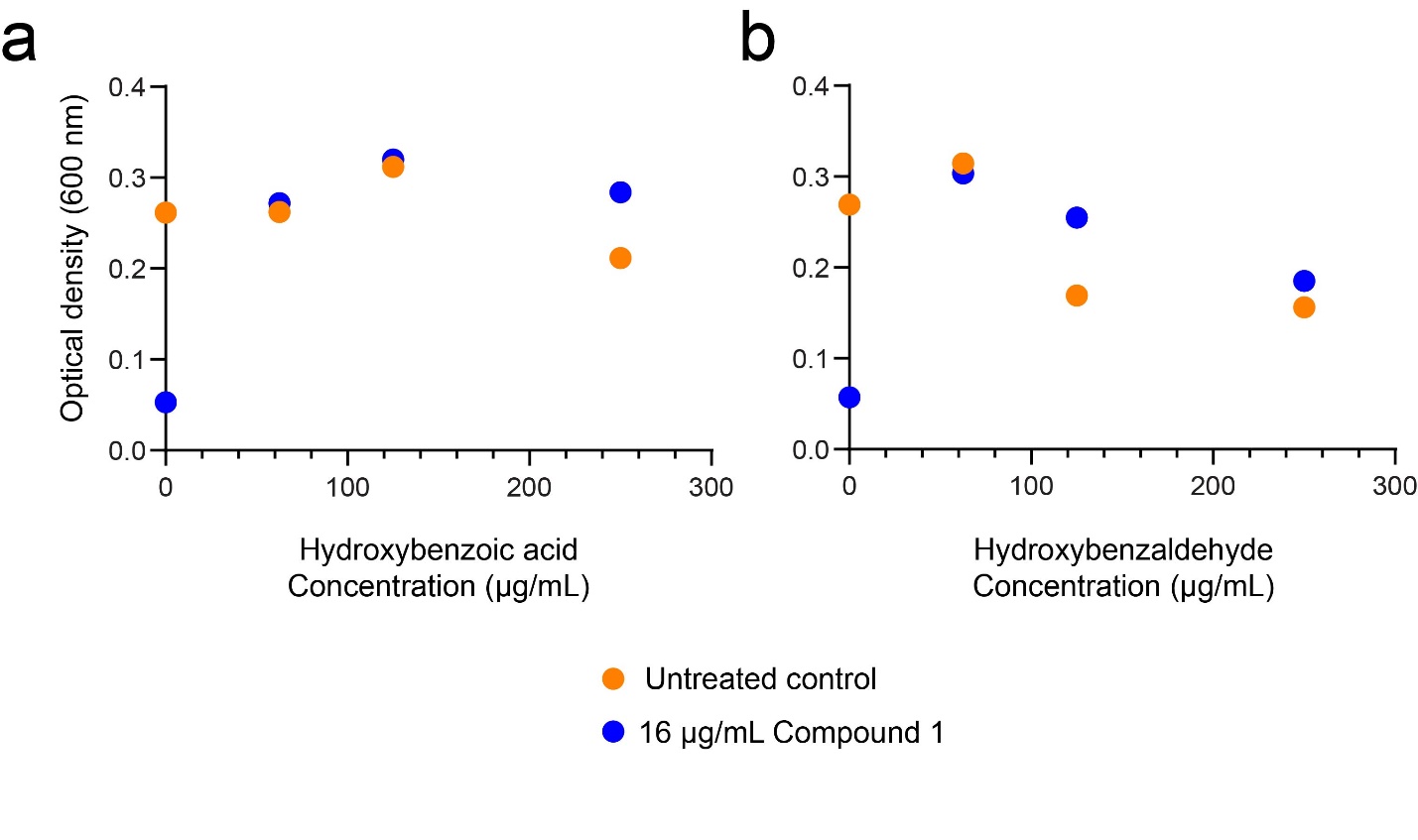
**

**Supplemental Figure 3.** *A. baumannii* homology model chorismate pyruvate-lyase (CPL) (Genbank: SST05187.1) in ribbon structure. (a) *A. baumannii* homology model is composed of α-helices (blue) and six antiparallel β-sheets (purple). (b) Superposition of *A. baumannii* homology model (pink) with crystal structure of *E. coli* CPL PDB:1FW9 (light blue), which is bound to natural substrate 4HB (gray). Superposition of the two models was performed by UCSF Chimera’s MatchMaker with cutoff point at 2Å. The calculated RMSD value of all superimposed residues is 0.780Å.


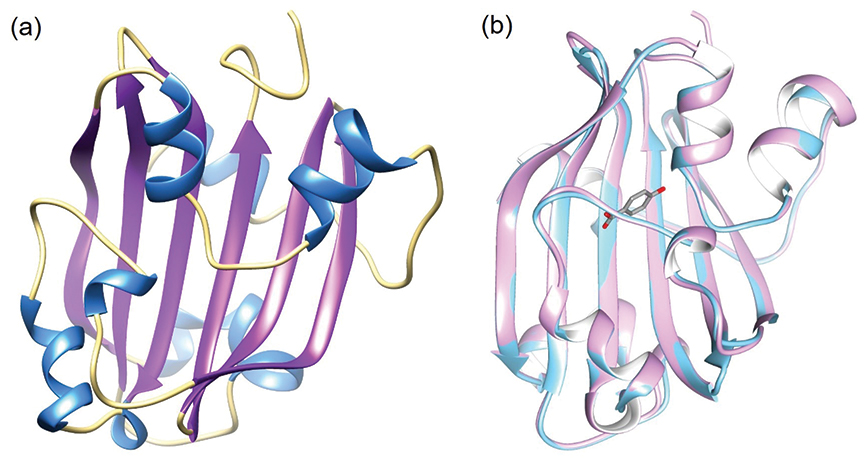


**Supplemental Figure S4.** Binding conformation of 4HB in *E. coli* CPL (PDB 1FW9) and compound **1** in A*. baumannii* (Genbank: SST05187.1) model. Van Der Waals forces (dotted lines) were observed between compound **1** and protein residues (yellow).


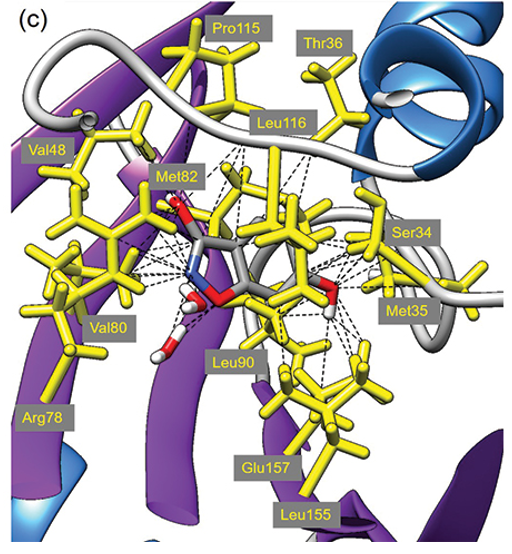


**Supplemental Figure 5.** Multiple sequence alignment of *E. coli* CPL (Uniprot P26602) with homologous CPL of four species of bacteria including *A. baumannii* (SST05187.1), *K. pneumoniae* (SSG33342.1), *P. aeruginosa* (VZT40144.1), and *S. marcescens* (WP_074054962.1). Dots indicate identical key residues and dashes indicate alignment gaps. Triangle (▲) and minus (**-**) symbols below the alignment indicate key residues involved in forming H-bonds and hydrophobic force with 4HB, respectively. The square () symbol indicates proposed residues involved in the binding of chorismate at the secondary site.

**
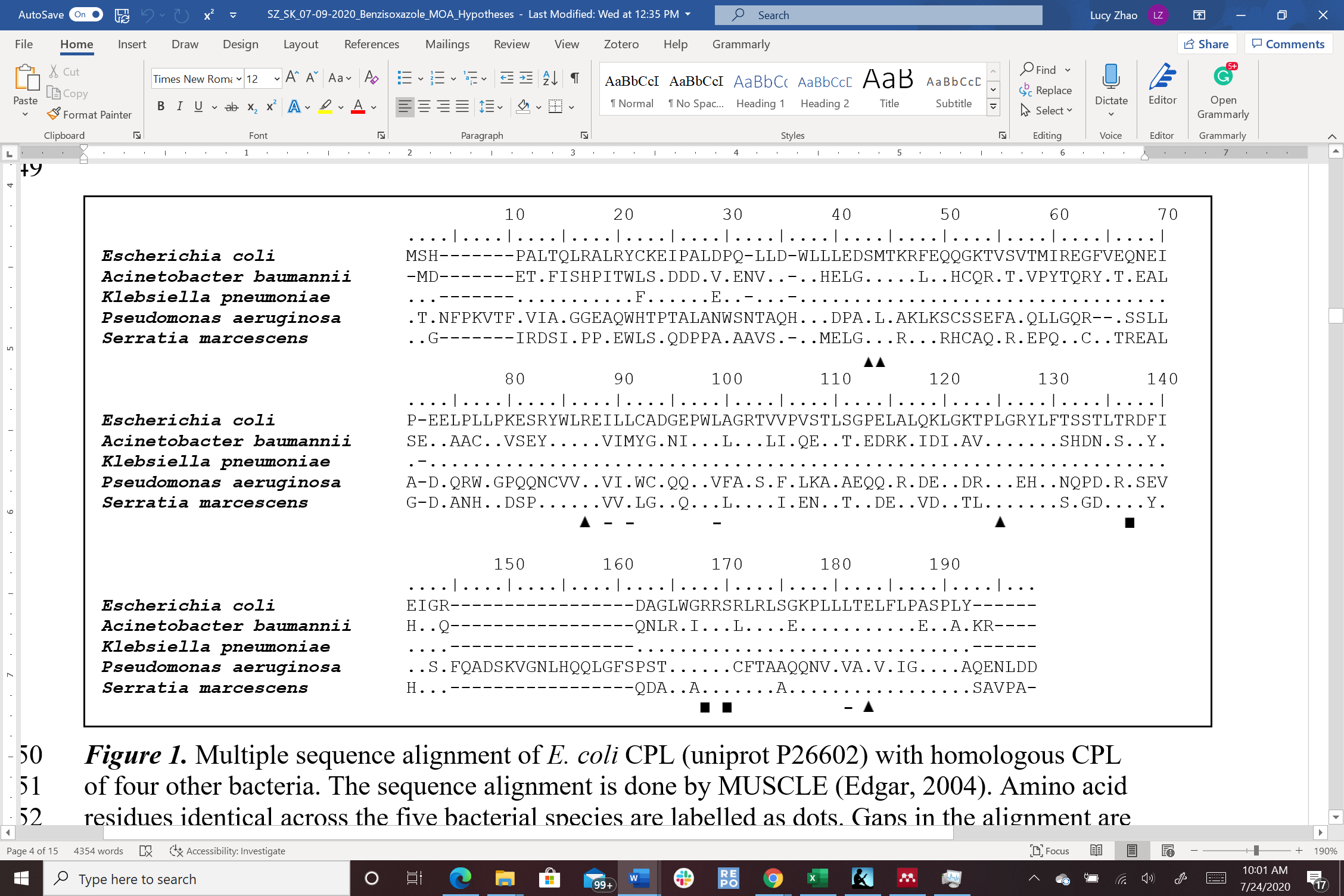
**

**Supplemental Figure 6.** Multiple sequence alignment of *E. coli* 4HB octaprenyltransferase (UniProtKB: P0AGD1.1) with two bacteria (*A. baumannii* WP_120755218.1; *P. aeruginosa* NP_254045.1) and two mammal (*M. musculus* UniProtKB: Q66JT7; human UniProtKB:Q96H96) species. Dots indicate identical key residues and dashes indicate alignment gaps. Triangle (▲) symbol represents below the alignment indicate those residues involved in 4HB binding to the active site.

**
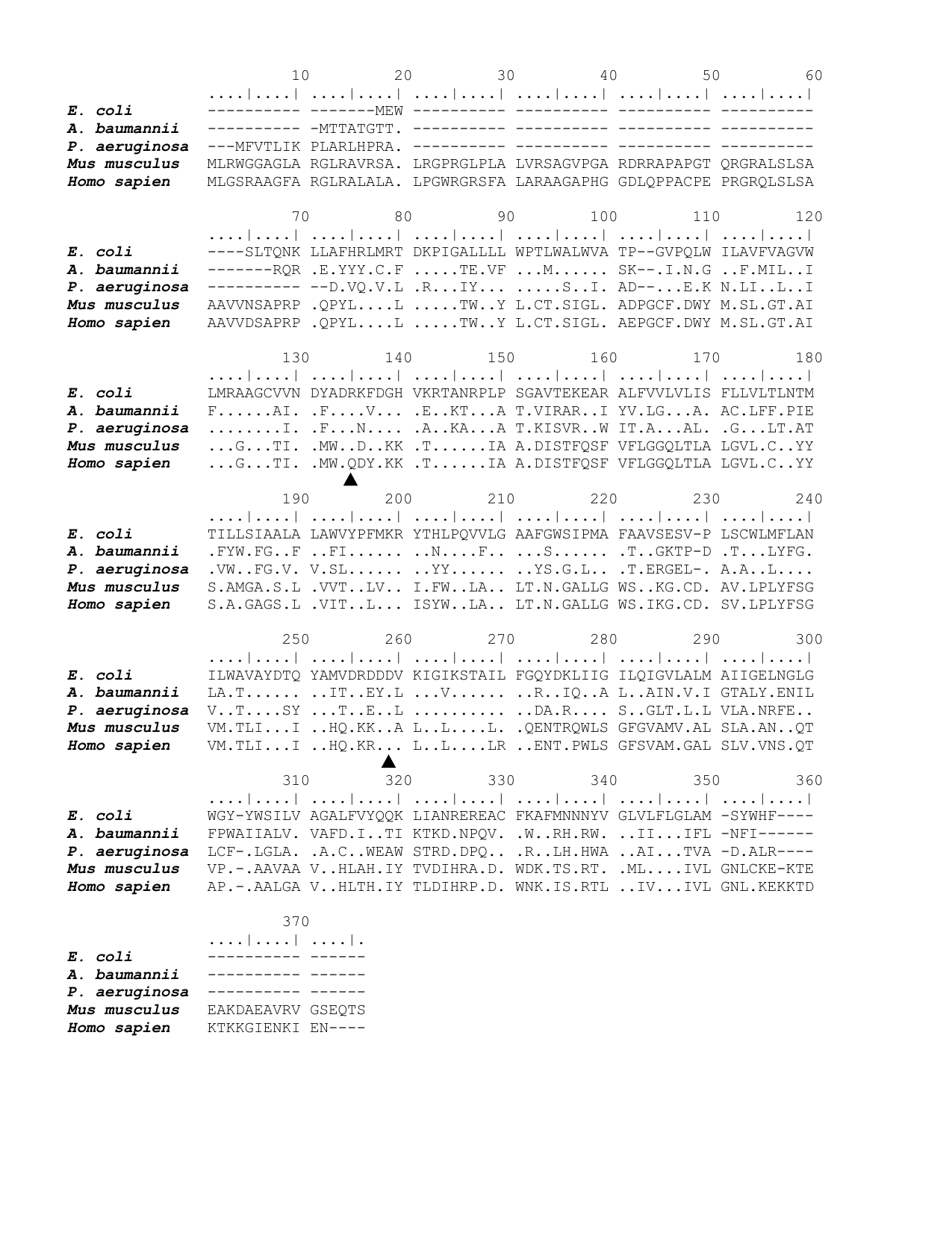
**

**Supplementary Tables**

**Supplemental Table 1.** Comparison of ^1^H and ^13^C-NMR data of **1**, synthetic 3,6-dihydroxy-1,2-benzisoxazole, and published 3,6-dihydroxy-1,2-benzisoxazole. Chemical shifts are referenced to residual methanol in CD_3_OD at *δ*_H_ 3.31 and *δ*_C_ 49.0.

|  | Synthetic 3,6-dihydroxy-1,2-benzisoxazole **(1)** | | | Published **1** | Isolated **1** |
| --- | --- | --- | --- | --- | --- |
| position | *δ*_C_, type | | *δ*_H_, (*J* in Hz) | *δ*_C_, type | *δ*_H_, (*J* in Hz) |
| 3 | 167.6, C | |  | 167.4, C |  |
| 3a | 108.2, C | |  | 108.1, C |  |
| 4 | 123.6, CH | | 7.50, d (8.6) | 123.6, CH | 7.49, d (8.6) |
| 5 | 114.5, CH | | 6.77, dd (8.6, 1.9) | 114.5, CH | 6.75, d (8.6) |
| 6 | 163.2, C | |  | 163.1, C |  |
| 7 | 96.2, CH | | 6.70, d (1.9) | 96.2, CH | 6.67, s |
| 7a | 166.9, C | |  | 166.8, C |  |
|  | |  |  |  |  |

**Supplementary Table 2.** MIC values for analog compounds **1-10** against *A. baumannii* strains. Values in bold indicate an inhibition was shown.

|  | Analog compounds | | | | | | | | | |
| --- | --- | --- | --- | --- | --- | --- | --- | --- | --- | --- |
|  | MIC (µg/mL) | | | | | | | | | |
| Strain | 1 | 2 | 3 | 4 | 5 | 6 | 7 | 8 | 9 | 10 |
| *A. baumanni* L1051 | **12.5** | >100 | >100 | >100 | **50** | >100 | >100 | >100 | >100 | >100 |
| *A. baumanni* NF-13382 | **6.25** | >100 | >100 | >100 | **50** | **100** | >100 | >100 | >100 | >100 |
| *A. baumanni* NR-17786 | **25** | >100 | >100 | >100 | **50** | **100** | >100 | >100 | >100 | >100 |
| *A. baumanni* ATCC 19606 | **50** | >100 | >100 | >100 | **50** | >100 | >100 | >100 | >100 | >100 |

**Supplemental Table 3.** Molecular docking of 4-HB and 3,6-dihydroxy-1,2-benzisoxazole in *E. coli* CPL (PDB 1FW9) and *A. baumannii* (GenBank: SST05187.1) homology model under solvated condition. AutoDock Vina’s docking score predicts the protein-ligand binding affinity.

| **Species** | **Ligand** | **Docking Score (kcal/mol)** |
| --- | --- | --- |
| *A. baumannii* | 4-HB | -5.9 |
| *A. baumannii* | 3,6-dihydroxy-1,2-benzisoxazole | -5.8 |
| *E. coli* | 4-HB | -7.3 |
| *E. coli* | 3,6-dihydroxy-1,2-benzisoxazole | -7.2 |
